# The Transmission of Disease by Sputum

**Published:** 1917-01

**Authors:** 


### THE TRANSMISSION OF DISEASE BY SPUTUM.

The campaign against tuberculosis not only has been effective against that disease, but it has also been of service, in a general sense, in promoting an increased attention to general hygiene, and therefore in a lessening of the modes by which infectious diseases are transmitted. Yet, it must be admitted that even the foremost prophylactic precept, that of the perniciousness of spitting, still is neglected in many places, and that its meaning by no means as yet is fully understood. As Dr. Wallace A. Manheimer (*Med. Rec.*, June 3, 1916, p. 997) points out, the delicatessen-store clerk who moistens his finger with saliva to pick up the piece of paper in which to wrap the butter is spitting on the paper, and, therefore, on the butter; the street-car conductor who wets his fingers and then gives you a transfer ticket is spitting on the paper slip and, therefore, on your hand.

The common habit of moistening the gum of postage stamps and envelopes with saliva, the author continues, has suggested the possibility of the transmission of disease through letters infected in that way. The evident contamination of letters when they are sealed and stamped and the frequent spreading of the saliva by the fingers and hand to other parts of the envelope suggest the importance of determining what the danger from this source really is. Letters are handled by the carriers and postal clerks almost immediately after their being mailed, and they are delivered to the addresses sometimes within a few hours, very often within but twelve or eighteen hours after being mailed.

Immediate contact of the hands with saliva and the subsequent introduction of the fingers into the mouth represents a much more dangerous mode of infection than, for example, the inhalation of dried sputum. The discharges from the mouth are responsible for almost as many diseases as are contracted from all other sources put together. Tubercle-bacilli transmitted in wet saliva are far more virulent than when dry and blown in the air. Wet or fresh sputum, when we consider the frequency with which it is spread about, represents the most dangerous material discharged by human beings. The pneumococcus, the diptheria bacillus, the germs of measles, scarlet fever, smallpox, whooping cough, epidemic meningitis, mumps, influenza, common colds, and other infections are more frequently transmitted through fresh sputum than in any other way. The author named refers to a recent case of syphilis contracted through counting paper money with fingers moistened with saliva, as strongly emphasizing the caution which should be observed from this source of infection.

All these things are so self-evident that they appear to be ludicrously simple. And, yet, even physicians offend against the most ordinary laws, not only of hygiene, but of prophylaxis; as witness the frequency with which one can see physicians moistening their fingers with their tongues to facilitate the turning of the leaves of a book. The present writer has watched one of the foremost tuberculosis physicians of our country do this same thing time and time again. True, he is not tuberculous and does not injure anybody; but it is, withal, a filthy habit and should be eschewed, if for no other than esthetic reasons.

It must be admitted that as physicians, who are supposed to be the teachers of the people in matters of health, we are not always consistent in our practicing what we preach, and it may be well to consider the warning voiced by Dr. Manheimer, in order to narrow still further the many ways in which infections may be transmitted.—*Clinical Medicine.*

